# Understanding Immune‐Driven Brain Aging by Human Brain Organoid Microphysiological Analysis Platform

**DOI:** 10.1002/advs.202200475

**Published:** 2022-07-31

**Authors:** Zheng Ao, Sunghwa Song, Chunhui Tian, Hongwei Cai, Xiang Li, Yifei Miao, Zhuhao Wu, Jonathan Krzesniak, Bo Ning, Mingxia Gu, Luke P. Lee, Feng Guo

**Affiliations:** ^1^ Department of Intelligent Systems Engineering Indiana University Bloomington IN 47405 USA; ^2^ Center for Stem Cell and Organoid Medicine (CuSTOM) Division of Pulmonary Biology Division of Developmental Biology Cincinnati Children's Hospital Medical Center Cincinnati OH 45229 USA; ^3^ University of Cincinnati School of Medicine Cincinnati OH 45229 USA; ^4^ Center for Cellular and Molecular Diagnostics Department of Biochemistry and Molecular Biology Tulane University School of Medicine New Orleans LA 70112 USA; ^5^ Harvard Institute of Medicine Harvard Medical School Harvard University Brigham and Women's Hospital Boston MA 02115 USA; ^6^ Department of Bioengineering Department of Electrical Engineering and Computer Science University of California at Berkeley Berkeley CA 94720 USA; ^7^ Department of Biophysics Institute of Quantum Biophysics Sungkyunkwan University Suwon Gyeonggi‐do 16419 South Korea

**Keywords:** aging, brain organoid, inflammaging, microfluidics, neuroimmune interaction

## Abstract

The aging of the immune system drives systemic aging and the pathogenesis of age‐related diseases. However, a significant knowledge gap remains in understanding immune‐driven aging, especially in brain aging, due to the limited current in vitro models of neuroimmune interaction. Here, the authors report the development of a human brain organoid microphysiological analysis platform (MAP) to discover the dynamic process of immune‐driven brain aging. The organoid MAP is created by 3D printing that confines organoid growth and facilitates cell and nutrition perfusion, promoting organoid maturation and their committment to forebrain identity. Dynamic rocking flow is incorporated into the platform that allows to perfuse primary monocytes from young (20 to 30‐year‐old) and aged (>60‐year‐old) donors and culture human cortical organoids to model neuroimmune interaction. The authors find that the aged monocytes increase infiltration and promote the expression of aging‐related markers (e.g., higher expression of p16) within the human cortical organoids, indicating that aged monocytes may drive brain aging. The authors believe that the organoid MAP may provide promising solutions for basic research and translational applications in aging, neural immunological diseases, autoimmune disorders, and cancer.

## Graphical abstract

The development of a human brain organoid microphysiological analysis platform to model immune–brain interaction is reported and the monocytes found from aged donors may drive brain aging.

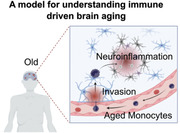

## 1 Introduction

Aging is a complex process with the accumulation of unrepaired molecular and cellular damage.^[^ ^1^ ^]^ During aging, chronic, sterile, low‐grade inflammation, named “inflammaging,” contributes to the pathogenesis of age‐related diseases such as Alzheimer's disease.^[^ ^2^, ^3^ ^]^ Aging of the immune system, or immunosenescence, drives systemic aging, including brain and other organisms.^[^ ^4^ ^]^ Taking one of the most important immune cell types in blood, bone marrow‐derived monocytes can infiltrate the aged and/or inflamed brain and get activated to secrete inflammatory molecules (e.g., TNF‐*α*), contributing to the inflammation in the aged brain.^[^ ^5^, ^6^, ^7^ ^]^ Tremendous interests have been attracted to studying immune‐driven brain aging, e.g., aged monocytes impacting brain aging and aging‐associated neural diseases, but little has been explored yet.^[^ ^8^, ^9^, ^10^ ^]^

Several models have been employed to investigate neuroimmune interaction for understanding inflammation and age‐related diseases. 2D in vitro cultures are simple, high throughput, and widely used to model the interaction of neuronal and immune cells. However, the 2D cultures lack the complex brain tissue architectures and neuroimmune microenvironments of an in vivo brain. Animal models have been demonstrated as excellent surrogates to study neuroimmunology in various physiological and pathological conditions. However, animal models are generally costly, time‐consuming, and cannot fully capture human biology due to their genomic and epigenomic differences from humans.^[^ ^11^, ^12^ ^]^ Superior to 2D cultures and animals, human brain organoids, 3D in vitro brain‐like tissues derived from human stem cells, can recapitulate some critical features of the human brain, including cellular identities, brain structures, microenvironments, and neural activities, bringing new opportunities to study neuroimmune interaction in aging and age‐related diseases.^[^ ^13^, ^14^, ^15^ ^]^ For example, human brain organoids have been deployed to model the microglia‐mediated neuroinflammation for understanding the etiology of Alzheimer's disease, substance use disorder, and other diseases.^[^ ^16^, ^17^, ^18^ ^]^ However, current organoids suffer from poor perfusion of oxygen/nutrients, variable reproducibility, and lack of immune components. Thus, there is an emergent and unmet need for the reproducible generation of standardized organoids with immune components to model neuroimmune interaction.

Recently, microfluidics and microfabrication technologies have been used for improving the organoid cultures and 3D in vitro cultures from the aspects of standardization, throughput, function, and utility.^[^ ^19^, ^20^, ^21^, ^22^, ^23^, ^24^, ^25^, ^26^, ^27^, ^28^ ^]^ For example, microfluidic droplets have been used to encapsulate cells into water‐in‐oil droplets or microgels for high throughput generation of uniformed human brain organoids and tumor spheroids.^[^ ^29^, ^30^, ^31^, ^32^ ^]^ Based on the development of acoustofluidics,^[^ ^33^, ^34^, ^35^, ^36^, ^37^ ^]^ our group has adapted this technology to assemble cells for the massive and scaffold‐free generation of standardized 3D cell cultures and assembloids.^[^ ^38^, ^39^, ^40^, ^41^, ^42^ ^]^ The microfabricated perfusion devices have been developed to perfuse oxygen, nutrients, and/or chemicals into organoids or 3D in vitro cultures to improve the generation, development, and maturation/vascularization of kidney organoids and brain organoids.^[^ ^43^, ^44^, ^45^, ^46^ ^]^ Pioneering efforts have also been made to develop automated microfluidic systems and mini‐bioreactors to generate and track many organoids and 3D cultures (e.g., gastrointestinal, tumor, and brain organoids) for high throughput screening applications.^[^ ^47^, ^48^, ^49^, ^50^, ^51^ ^]^ Despite current microfluidic devices and systems having significantly improved the contemporary culture of organoids, there is still an unmet need to develop user‐friendly and scalable platforms that can reproducibly generate standardized organoids with immune components and allow the time‐lapse imaging of neuroimmune interaction for studying aging and age‐related diseases.

Herein, we developed an innovative organoid microphysiological analysis platform (MAP) to generate one standardized organoid per device and enable its interactions with immune cells. Our MAP has several advantages. 1) Our MAP design allows us to confine a developing organoid into the pancake shape as well as perfuse oxygen nutrients to reduce hypoxia and necrosis. 2) Our method can mimic blood vessels to flow immune cells (e.g., monocytes) toward organoids for studying immune–organoid interaction. 3) In contrast to the traditional organoid culture methods (well‐plates on a rocking machine, or bulk bioreactors), our technology immobilizes one organoid per device to avoid the organoid fusion as well as improve the standardization of organoid cultures. 4) Our 3D printed devices are simple, low‐cost, user‐friendly, and compatible with common labware. They can be easily adapted with the current organoid protocol for the high throughout investigation of immune–organoid interaction. We used this platf to model immune‐driven brain aging using primary human monocytes as a proof‐of‐concept. Interestingly, our results indicated that aged monocytes may induce an aging‐related phenotype inside human brain organoids (higher expression of p16 and p21 within the organoids) without external genetic manipulation.

## 2 Results

### 2.1 Understanding immune‐driven brain aging using human brain organoid MAP

**Figure 1:**
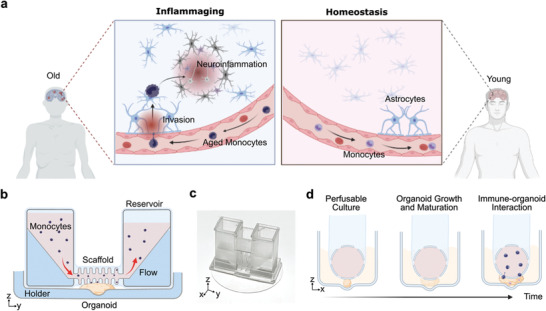
Understanding immune‐driven brain aging using human brain organoid microphysiological analysis platform (MAP). a) Schematics showing the 3D printed devices for modeling immune‐driven brain aging by culturing and analyzing the interaction of primary monocytes (from young and aged donors) and human brain organoids. b) The schematics of MAP consist of 3D printed devices within a well‐plate and a single device with an organoid holder and a tubular perfusable scaffold connected with two medium reservoirs. c) Picture of an MAP device. d) Schematics show the experimental setup of the MAP device to study immune–organoid interactions.

To understand immune‐driven brain aging (**Figure** **1**a), we developed an organoid MAP to investigate human brain organoid interaction with monocytes isolated from old and young donors in a standardized and user‐friendly manner. Our MAP device (design details in Figure S1, Supporting Information) consists of two components (Figure 1b,c): 1) a hollow and meshed tubular perfusable scaffold connected with two medium reservoirs for supporting the organoid growth along the tubular scaffold surface, perfusion of oxygen and nutrients, and mimicking blood vessels to flow in immune cells (e.g., monocytes) toward organoids, and 2) an organoid holder on a thin glass coverslip for holding the organoids within well‐plates. A developing organoid can grow into a pancake‐shape structure within the confined space between the tubular perfusable scaffold and the organoid holder (within 400 µm). Once combined with rocking flows, our MAP could minimalize necrosis and hypoxia of the on‐chip cultured organoids, facilitate the noninvasive introduction of immune cells, and enable the time‐lapse imaging of dynamic immunocyte–organoid interaction (Figure 1d). Due to the simplicity of medium exchange, our MAP could be widely adapted to culture different types of organoids.

### 2.2 On‐Chip Perfusable Culture of Human Cortical Organoids

**Figure 2:**
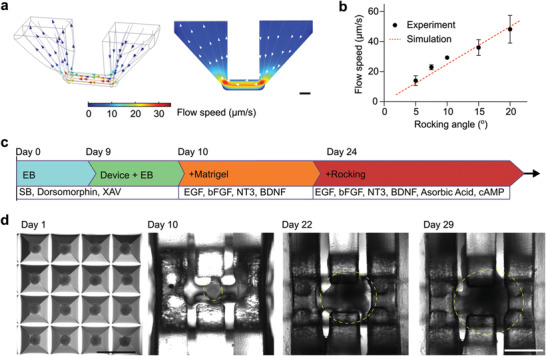
On‐chip culture of human cortical organoids. a) The simulation results indicate the 3D distribution (left) and side view (right) of flow profiles within the device. b) The experimental results describe the flow speed under different rocking angles (rocking frequency = 0.1 rpm). c) The illustration shows the protocol and time of the on‐chip culture of human cortical organoids. d) Top‐down view images of organoids during the differentiation and on‐chip culture process (organoids in yellow dashed circles). Scale bar: 1 mm.

Before applying our MAP for the organoid culture experiment, we generated and optimized the workflow to generate and culture human cortical organoids on‐chip. Through the simulation, our results described the 3D distribution (**Figure** **2**a, left) and the side view (Figure 2a, right) of flow profiles within the MAP device (rocking angle = 15, and rocking frequency = 0.1 rpm, Discussion S1, Supporting Information). With the theoretical prediction, we further measured the dependence of the flow speed on the rocking angle (dots in Figure 2b, with rocking frequency = 0.1 rpm), which matched our simulation result (a red dashed line in Figure 2b). We adopted a culture protocol to generate human cortical organoids (hCOs) on‐chip (Figure 2c). Briefly, embryonic bodies (EBs) with a diameter of ≈300 µm were assembled from human embryonic stem cells (hESC) (WA01, Wicell) with SB‐431542 and XAV‐939 to inhibit TGF and Wnt/*β*‐catenin signaling for neural induction and patterning. The EBs were then transferred to the organoid holder of the devices within a well‐plate on day 9, embedded in Matrigel in situ, and cultured inside the device for 15 days till forming a hollow structure surrounding the mesh tubular scaffold (Figure 2d). Various growth factors, such as BDNF, EGF, or FGF, were added to the medium for guiding the differentiation, maturation, and survival of neurons. On day 24, the devices within the well‐plate were finally transferred to a rocking platform to generate inner lumen fluid flow. Meanwhile, ascorbic acid and cAMP were added to support the differentiation of neural progenitor cells into mature neurons and support organoid growth.

### 2.3 Characterization of Human Brain Organoid MAP

**Figure 3:**
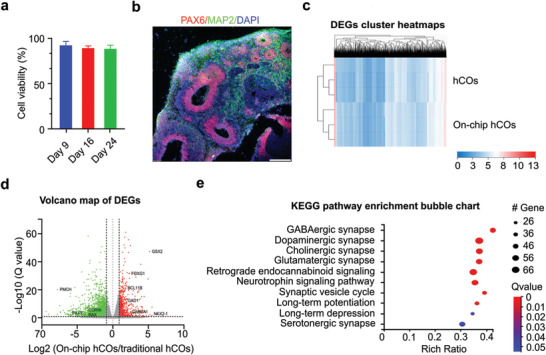
Characterization of on‐chip cultured organoids. a) Viability of on‐chip cultured human cortical organoids (on‐chip hCOs) during long‐term culture. b) Cross‐section staining showing VZ/SVZ (PAX6 and MAP2 neuron) distribution inside the on‐chip hCO on day 29. c) Cluster heatmaps of differentially expressed genes (DEGs) in the on‐chip hCOs and the conventional human cortical organoids (hCOs). d) Volcano map of DEGs with red (increased expression) and green (decreased expression) dots. e) KEGG pathway enrichment bubble chart showing high‐level functions and utilities in the on‐chip hCOs than the conventional hCOs. Scale bar: 500 µm.

We characterized the development of the on‐chip cultured hCOs using viability assays, immunostaining, and sequencing approaches. We first used live/dead staining to test the viability of on‐chip cultured hCOs on days 9, 16, and 24, respectively. As expected, with the support of medium perfusion, on‐chip cultured hCOs maintained very high viabilities during a prolonged culture time (**Figure** **3**a). We then confirmed the proper development and differentiation of on‐chip cultured hCOs using PAX6 (neural progenitor) and MAP2 (neuron) staining. We found that the on‐chip cultured hCOs successfully developed with proper ventricular/subventricular zones (VZ/SVZ) indicated by PAX6 staining as well as cortical plates indicated by MAP2+ neurons surrounding VZ/SVZ. (Figure 3b). We further compared gene expression of on‐chip cultured hCOs with conventional hCOs (cultured on an orbital shaker) using bulk RNA sequencing. Three on‐chip cultured hCOs and three conventional hCOs were used to determine the change in gene expression related to neural development. To visualize the differentially expressed genes in our on‐chip cultured and conventional hCOs, we first performed a hierarchical clustering analysis of the gene expression profiles. By unsupervised clustering of the gene expression profiles of the three conventional hCOs and three on‐chip hCOs, we found that the gene expression profiles of three conventional hCOs clustered together and the three on‐chip hCO gene expression profiles clustered together (Figure 3c), this indicated that the on‐chip culture‐induced significantly changes in hCO expression profiles, making them phenotypically distinct from convention hCOs. Upon detailed examination of differentially expressed genes, we found that genes related to forebrain development (GSX2, FOXG1, and NKK2‐1),^[^ ^52^ ^]^ deep layer cortical neuron development (BCL11B),^[^ ^53^ ^]^ and GABAergic neuron development (GAD1 and GABRA1)^[^ ^54^ ^]^ were significantly enriched in the on‐chip cultured hCOs than conventional hCOs. Meanwhile, non‐forebrain fate markers such as RAX and PMCH,^[^ ^52^, ^55^ ^]^ frequently found in the hypothalamus, were lower expressed in on‐chip cultured hCOs than conventional hCOs (Figure 3d). These results indicated that the on‐chip culture could facilitate forebrain fate commitment in hCOs, which is consistent with our previous report.^[^ ^17^ ^]^ We then further performed a Kyoto Encyclopedia of Genes and Genomes (KEGG) pathway enrichment analysis to identify significant pathways impacted by on‐chip culture. We found that the differentially expressed genes were significantly enriched in diverse synaptic development pathways such as GABAergic, dopaminergic, cholinergic, and glutamatergic synapses pathways (Figure 3e). Overall, these results show that our MAP devices could facilitate forebrain fate commitment, neuron, and synapse maturation in hCOs.

### 2.4 Investigation of Monocyte Infiltrating Organoid Using Human Brain Organoid MAP

**Figure 4:**
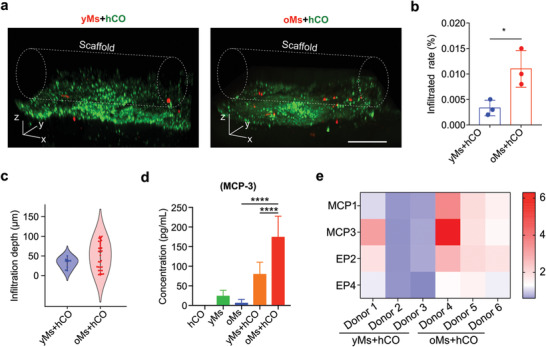
Characterization of monocyte inflitrating organoid. a) Comparison of infiltration capacity of monocytes isolated from old donors (>60‐year‐old) into on‐chip cultured human cortical organoid (oMs+hCO) with monocytes isolated from young donors (20 to 30‐year‐old) into on‐chip cultured human cortical organoid (yMs+hCO). Scale bar: 200 µm. b) Quantification of infiltrated monocytes in “yMs+hCO” and “oMs+hCO” groups, *n* = 3. c) Violin plot showing infiltration depth distribution of monocytes into the organoids in both yMs+hCO and oMs+hCO groups. d) ELISA analysis of MCP‐3 concentrations in supernatant isolated from hCO, yMs, oMs, yMs+hCO, and oMs+hCO cultures, *n* = 6. e) The plot of gene expression change (2^−ΔΔCt^) heatmap of the “yMs+hCO” and “oMs+hCO” groups on day 29 under the same experimental conditions, *n* = 3.

In the aged brain, monocytes may have a greater capacity to infiltrate the brain on the brain‐blood barrier (BBB) during injury or neurological disorders.^[^ ^56^ ^]^ Particularly, animal experiments showed that aged mice harbor expanded CCR2+ monocytes postinjury/chronic inflammation that can infiltrate the brain based on MCP1/CCR2 chemotaxis signaling.^[^ ^57^ ^]^ Postinfiltration, monocytes may induce proinflammatory responses in neighboring glial cells such as astrocytes. To investigate the differential infiltration capacity of young and aged monocytes into hCOs, we perfused the primary monocytes isolated from peripheral blood mononuclear cells (PBMCs) into the on‐chip cultured hCOs. We collected the old monocytes (oMs) from the PBMCs from the aged (>60 years old) donors and obtained young monocytes (yMs) from the young (20–30 years old) donors. After perfusing oMs and yMs into devices separately, we recorded the monocyte infiltrating organoid on‐chip (**Figure** **4**a). We found the infiltrated cell number of oMs was ≈3 times compared to that of yMs at 24 h postperfusion (Figure 4b), with higher infiltration depth (Figure 4c) indicating oMs have significantly higher infiltration capacity than yMs. Upon further enzyme‐linked immunoassay (ELISA) analysis of the supernatant from hCO alone, yMs, oMs, hCO coculture with yMs, (yMs+hCO) and hCO coculture with oMs (oMs+hCO), we found that one key chemokine mediating monocyte chemotaxis: MCP‐3, was significantly upregulated in the oMs and hCO coculture systems, but not in oMs culture alone or hCO culture alone (Figure 4d). Upon further quantitative reverse transcription polymerase chain reaction (qRT‐PCR) analysis, we also found that the cocultured oMs and hCO (oMs+hCO) expressed higher levels of monocyte chemoattractant genes such as MCP1 and MCP3 than the cocultured yMs and hCO (yMs+hCO) (Figure 4e). This indicated that the increased infiltration capacity of oMs could also be attributed to MCP1/MCP3/CCR2 signaling axis, which might be secreted by activated astrocytes in hCOs.^[^ ^58^, ^59^ ^]^

### 2.5 Investigation of Monocyte‐Driven Brain Aging Using Human Brain Organoid MAP

**Figure 5:**
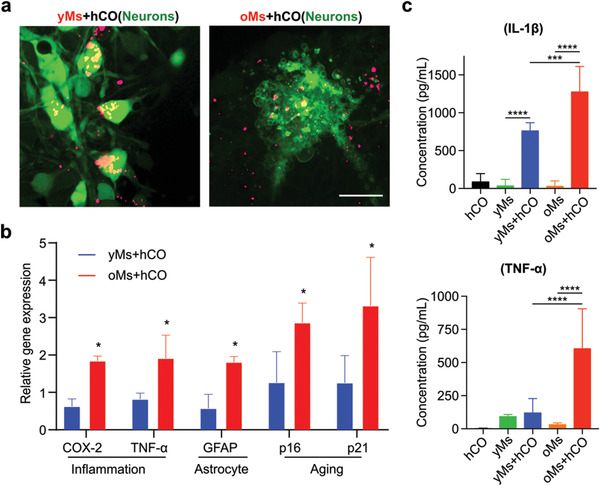
Characterization of monocyte‐driven aging phenotypes. a) Confocal images of neuron morphology adjacent to infiltrated monocytes isolated from old donors (>60‐year‐old) into on‐chip cultured human cortical organoid (oMs+hCO) with monocytes isolated from young donors (20 to 30‐year‐old) into on‐chip cultured human cortical organoid (yMs+hCO). Scale bar: 20 µm. b) Comparison of proinflammation genes (COX‐2 and TNF‐*α*) and senescence genes (p16^INK4a^ and p21^CIP1^) in monocytes within oMs+hCO and yMs+hCO cultures on day 29. 2^−ΔΔCt^ calculated as “delta Ct” (∆∆Ct) of GAPDH and target gene normalized against on‐chip cultured human cortical organoid only on the same day, *n* = 3. c) Characterization of IL‐1*β* and TNF‐*α* concentrations in supernatant isolated from hCO, yMs, oMs, yMs+hCO, and oMs+hCO cultures, *n* = 6.

Cellular senescence and neuron apoptosis are the hallmarks of brain aging, as indicated by senescence markers such as p16. The expression of p16 is driven by various genetic and environmental factors such as inflammation. Thus, we sought to investigate the effect of monocyte infiltration into hCOs. Notably, the differential changes that young and old monocytes bring to hCOs, regarding inflammation, aging markers as well as neuron morphology. By staining neurons in hCO via calcium dye (Cal520, green), we found that neurons adjacent to infiltrated oMs within the oMs+hCO group (prelabeled with DiL membrane dye, red) show an apoptotic morphology (**Figure** **5**a), which was not observed in the yMs+hCO group. By qRT‐PCR analysis of on‐chip cocultured monocytes and hCO, we discovered an upregulation of prostaglandin E2 (PGE2), COX‐2, and TNF‐alpha (Figure 5b), which are age‐associated macrophage related proinflammation genes,^[^ ^60^, ^61^ ^]^ as well as upregulation of GFAP, as an indication of astrocyte activation within the oMs+hCO group.^[^ ^62^, ^63^ ^]^ Interestingly, we also found a significantly higher expression of aging‐related markers including p16 and p21 in the oMs+hCO group. We further confirmed that oMs induced proinflammatory responses in hCOs by analyzing the proinflammatory cytokines: IL‐1*β* and TNF‐*α* in the culture supernatants. Both cytokines were elevated in oMs and hCO coculture systems but not in oMs 2D culture alone. Additionally, both elevations were more profound in oMs infiltrated hCOs as compared with yMs infiltrated hCOs. These results indicated a potential mechanism for infiltrated monocytes from aged individuals to induce proinflammatory responses in hCOs as well as induce p16 expression inside hCOs to promote brain aging. Although a detailed mechanism must be elucidated, our findings shed light on a potential approach to inducing aging phenotype inside hCOs without external genetic manipulation.

## 3 Discussion

Aging is a systemic, multiorgan process, often accompanied by chronic and low‐grade inflammation known as inflammaging.^[^ ^64^ ^]^ This chronic inflammation, together with the senescence‐associated secretory phenotype of the peripheral immune system, could future drive the aging of the solid organs.^[^ ^65^ ^]^ Senescent organs, in turn, recruit more inflammatory immunocytes, which further catalyze this detrimental cycle.^[^ ^62^ ^]^ Brain, protected by the BBB, is often considered an immune‐privileged organ. However, during aging, BBB could often become permeable to immunocytes as adhesion molecules on BBB often get upregulated, and tight junctions got compromised during aging.^[^ ^66^ ^]^ Moreover, enlarged perivascular space often accompanies aging, leading to a reduction in blood flow, further promoting immunocytes adhesion and infiltration.^[^ ^67^ ^]^ Such immunocyte infiltration will likely lead to a cascade of proinflammatory responses and often preludes cognitive decline as well as, in some cases, neural degeneration.^[^ ^68^ ^]^

To recapitulate this process in vitro and interrogate the roles of aged immunocytes in driving brain aging, here we presented a novel human brain organoid MAP to model theshe neuroimmune interaction in aging. By integrating an organoid scaffold and rocking flow, our MAP allows the generation of standardized human cortical brain organoids without hypoxia and necrosis. The on‐chip culture organoids showed significant forebrain fate commitment compared to the traditional culture. Additionally, due to the noninvasive incorporation of immune cells into organoids with unique tubular scaffold and rocking flow, our MAP supports the investigation of primary immune–organoid interaction on‐chip, recapitulating the key features and processes of neuroimmune interaction in the aged brain where BBB becomes compromised.

Utilizing this system, we discovered that monocytes isolated from aged individuals could exert a unique phenotype on young brain organoids. This includes increased MCP‐3 chemokine to attract more immunocytes, increased prostaglandin E2 signaling, as well as increased proinflammatory cytokine secretion. Additionally, neurons surrounding infiltrated aged monocytes also show an apoptotic morphology, further supporting the hypothesis that an aged peripheral immune system could potentially contribute to brain aging and in severe cases, neural degeneration.

One limitation of our current MAP model is that the organoids were derived from hESCs that biologically have long telomeres, which do not reflect the senescent phenotypes of an aged brain. Further development of the model could incorporate genetic modeling of organoid aging by overexpressing aging‐related genes such as progranulin in organoids. Additionally, experiments in this study were performed on 45‐day‐old organoids. Prolonged culture on organoids may promote further differentiation and potentially senescence phenotypes. Future studies are underway to interrogate the effect of culture time on organoid senescence phenotypes. Additionally, in this model, we presumed the scenario of a compromised BBB, where immunocytes come in direct contact with brain parenchyma. Incorporating a functional BBB in our model could further perfect this system to study how immunocytes interact with and even contribute to the breakdown of BBB in aging. To conclude, our MAP is simple, scalable, cost‐effective, and compatible with well‐plates and lab settings. We, therefore, believe that our technology provides avenues to study immune‐driven brain aging and restoring brain homeostasis in aging, intending to improve neurodegenerative functions.

## 4 Experimental Section

### Design and Fabrication of 3D Printed Devices

The MAP devices were designed using AutoCAD software and fabricated using a 3D printer (Form 3B, Formlabs). The detailed design of the MAP devices was described in Figure S1 of the Supporting Information.

### Simulation and Experimental Measurement of Flow Profiles

The flow profiles of the MAP device were simulated using Comsol Multiphysics (COMSOL group, details in Discussion S1 and Figure S2, Supporting Information). Briefly, the rocking flow was affected using the “laminar flow” physics module, and the liquid‐scaffold boundary was considered nonslip. To model the fluid movement, “moving mesh” was applied to the fluid domain, and the top air–liquid interface was set as a “free surface” model. Half of the model was simulated with symmetric boundary conditions, and quantification results were obtained using the 2D model to save the computational energy. The experimental measurement of the flow speed was conducted by tracking and calculating dye transferring with the device under five different rocking angles (details in Discussion S2 and Figure S3, Supporting Information). Briefly, the device was placed on the rocker at a neutral position. 30 µL of culture medium was added to both sides of the medium reservoir, with one side containing 0.83 mg mL^−1^ of Rhodamine B. The flow induced by the rocking cycle was videotaped at 30 frames per second with a fixed‐positioned camera secured on the rocker with a tripod. Video frames were analyzed with ImageJ. The flow speed was calculated by timing the moving dye front passing through a predetermined distance (3 mm) within the scaffold device.

### Culture of WA01 Cells and THP‐1 Cells

The hESC line (WA01, WiCell) was maintained with mTeSR plus medium (Stemcell Technologies) on 6 well plates coated with Matrigel (Corning). The WA01 cells were passaged every 5–7 days with a medium change every other day and generated organoids under passage 42. The THP‐1 (ATCC TIB‐202) cells, peripheral blood‐derived monocytes, were cultured in RPMI 1640 (Gibco) supplemented with 10% fetal bovine serum (Gibco), 0.05 mm 2‐mercaptoethanol (Sigma), and 100 U mL^−1^ penicillin/streptomycin (Gibco). Cells were maintained at 37 °C, 5% CO_2_, and passaged before the cell concentration reached 1 × 10^6^ cells mL^−1^. Human embryonic cell line WA01 was obtained from WiCell research institute, under the agreement between Indiana University and WiCell research institute, all experiments were performed under the guidance of this agreement.

### Differentiation Protocol of Human Cortical Organoids

Culture of hESCs were dissociated into single cells using ReLeSR (Stemcell Technologies) treatment. Single cells were plated to ultralow attachment 96 well plate (Corning) with AggreWell EB Formation Medium (Stemcell Technologies) with 10 µm Y‐27632 (SelleckChem) (9000 cells per well). After 24 h, the medium was substituted by cortical organoid medium (1× DMEM/F12; 15% KOSR; 1× GlutaMax; 1× MEM‐NEAA; 1× N2 supplement; 1× N2 supplement; 1 × 2‐Mercaptoethanol; 1× Penn/Strep) supplemented with 1 µm SB‐431542, 2 µm Dorsomorphin and 2 µm XAV939 for 9 days. Next, the aggregates were embedded in Matrigel and cultured in the medium (0.5× DMEM/F12; 0.5× Neuralbasal; 1× N2; 1× MEM‐NEAA; 1× GlutaMax; 2.5 µg mL^−1^ Insulin; 1× b‐mercaptoethanol; 1× Penn/Strep; 1 × 10 ng mL^−1^ EGF, 10 ng mL^−1^ FGF‐2, 10 ng mL^−1^ NT3, and 10 ng mL^−1^ BDNF) supplemented with 1× B27 without vitamin A for 14 days. Then the aggregates were maintained in the medium (supplemented with 1× B27 with vitamin A, 0.2 mm ascorbic acid, 0.2 mm cAMP, and 1% Matrigel) with a medium change every other day. The composition details and medium change timelines are provided in Table S1 of the Supporting Information.

### Cell Viability Test

To measure long‐term cell viability, hCOs were stained with a LIVE/DEAD Viability/Cytotoxicity Kit (Invitrogen). Samples were incubated with a carboxyfluorescein succinimidyl ester and ethidium homodimer‐1 (EthD‐1) mixture for 30 min at 37 °C. After washing the samples twice with the fresh medium, the fluorescence microscopy of the two different dyes was visualized by an Olympus IX83 inverted motorized microscope.

### Bulk Cell Sequencing

RNA was extracted from hCOs using Qiagen RNeasy plus mini kit. The RNA samples were sent to BGI Americas for RNA‐sequencing analysis. The RNA‐seq data were analyzed and visualized using Dr. Tom's data visualization software.

### Isolation of Monocytes from Human PBMCs

To use primary monocytes, human PBMCs were purchased from Innovative Research where they acquire the samples from partner FDA‐approved collections sites. PBMCs were isolated from human buffy coats of six different healthy young (*n* = 3, <35) and elderly (*n* = 3, >65) donors. The human buffy coats were first spun sown at 300 g for 10 min. To remove RBCs, the supernatant was discarded, and 5 mL of ACK lysing buffer was added to the pellet, which was shacked for 5 min. After adding 20 mL of HBSS, the tube was centrifuged at 300 g for 10 min. RBCs removal process was repeated until RBCs were negligible in the cell pellet. Monocytes were isolated from PBMCs using the Classical Monocyte Isolation Kit according to the manufacturer's protocol (Miltenyi Biotec GmbH) with MS Column (Miltenyi Biotec GmbH). Typically, 5 × 10^4^ monocytes per milliliter of whole blood were obtained.

### On‐Chip Coculture of Monocytes and Organoids

The hCOs of day 9 were carefully collected and washed in the pre‐warmed COM I with a cupped tip. After aspiration of the surrounding medium, organoids were carefully transferred to the center chamber with 6 µL of the Matrigel and incubated in a 37 °C incubator for 30 min. The scaffold was inserted onto the center chamber with an additional fresh medium followed by incubation. The device was then maintained in the COM II until day 24 with a medium change every other day. When changed to COM III, the organoids on‐chip was cultured onto a platform rocker (Infinity rocker, Next Advance) with 1 cycle min^−1^ in the incubator. To infiltrate monocytes into the device, a total of 1 × 10^5^ cells of monocytes were suspended in 20 *μ*L of COM III and added to the on‐chip cultured hCOs of 6 weeks old (45 days). After 2 h of incubation, the upper component of the device with an additional medium was inserted into the hCOs. For control hCOs, the hCOs of day 9 were transferred to an ultralow adherence 24 well plate and kept on an orbital shaker set at 60 rpm. Medium change was kept on the same schedule as the on‐chip cultures.

### Immunofluorescence Staining

Immunofluorescence staining was performed on sectioned samples directly on slides to characterize on‐chip cultured hCOs. The samples were washed twice with 1× PBS to remove the O.C.T. After antigen retrieval for 15 min with 3 n hydrochloric acid (HCl) treatment, the sections were rinsed twice with 1× PBS and then treated with 0.3% Triton‐X100/5% normal goat serum in PBS for 1 h. Next, primary antibodies were added to the slides in a diluted antibody solution and incubated overnight at 4 °C within a dark, humidified slide box. The samples were rinsed three times with 1× PBS and incubated with secondary antibodies at RT for 1 h, then the slides were washed three times with FBS. Coverslips were mounted with ProLong Gold Antifade Mountant with DAPI (Invitrogen). Detailed information on primary and secondary antibodies can be found in Table S2 of the Supporting Information.

### qRT‐PCRAnalysis

The Gene expression profile of hCOs and on‐chip cultured hCOs was evaluated by qRT‐PCR. In brief, organoids were first washed twice with PBS and lysed using RNeasy Plus Mini Kit (Qiagen) for RNA extraction. RNA was reverse transcribed to ds‐cDNA using the qScript cDNA synthesis kit (Quanta Biosciences). Applied Biosystems Power SYBR Green PCR Master Mix (Thermo Fisher) was used to carry out the real‐time qPCR. Detailed information on primers can be found in Table S3 of the Supporting Information. ΔΔCt method was performed to calculate relative expression (−ΔΔCt), the delta Ct value of target gene between organoid and organoid chips, both normalized against Ct value of the housekeeping gene: GAPDH. Each qPCR reaction was tested in triplicates, and three organoids/brain‐in‐a‐chips were analyzed for each group. The means of Ct values are represented. Samples as “not detected” were denoted as a Ct value of 40.

### ELISA

The cytokine production of monocytes was measured by ELISA. A total of 1 × 10^5^ cells of monocytes were added to each hCO of 6 weeks old with 100 µL of cortical organoid media III. After culturing in a 37 °C incubator for 24 h, 90 µL medium was aspirated into an Eppendorf tube and centrifuged at 300 g for 10 min. After centrifugation, 80 µL of the supernatant was diluted to 250 µL. Concentrations of MCP‐3, IL‐1*β*, and TNF‐*α* were measured by using Quantikine ELISA Human CCL7/MCP‐3 (R&D systems), Human IL‐1*β* ELISA Kit (Invitrogen), and Human TNF‐*α* ELISA Kit (Invitrogen), respectively. For each ELISA measurement, 50 µL of the diluted culture medium was used. The results were read by a Synergy H1 plate reader (BioTek). The standard curve was used to calculate the concentration of cytokines.

### Confocal Imaging

For confocal imaging, the monocytes were labeled with Vybrant DiL (red) (Invitrogen) at 1:1000 dilutions at 37 °C for 30 min before seeding onto hCOs. A total of 1 × 10^5^ cells of labeled monocytes were added to each hCO of 6 weeks old with 2 mL of BrainPhys Imaging Optimized medium (Stemcell Technologies). After coculturing in a 37 °C incubator for 65 h, the hCOs were stained with Cal520 calcium dye (Abcam) at 1:1000 dilutions at 37 °C for 30 min and then washed twice with 1× PBS. Pictures of neuron damage were taken using an Olympus OSR spinning disk microscope at 60× oil objective lens.

### Statistical Analysis

The statistic comparison of each group was performed via t‐test with GraphPad Prism 7. The statistical significance of differences in values is denoted as following *: *p* < 0.05, **: *p* < 0.01, ***: *p* < 0.005, ****: *p* < 0.001.

## Conflict of Interest

The authors declare no conflict of interest.

## Supporting information

Supporting Information

## Data Availability

The data that support the findings of this study are available from the corresponding author upon reasonable request.

## References

[1] T. B. L. Kirkwood , Cell 2005, 120, 437. 15734677 10.1016/j.cell.2005.01.027

[2] C. Franceschi , M. Bonafã , S. Valensin , F. Olivieri , M. De Luca , E. Ottaviani , G. De Benedictis , Ann. N. Y. Acad. Sci. 2000, 908, 244. 10911963 10.1111/j.1749-6632.2000.tb06651.x

[3] P. L. Minciullo , A. Catalano , G. Mandraffino , M. Casciaro , A. Crucitti , G. Maltese , N. Morabito , A. Lasco , S. Gangemi , G. Basile , Arch. Immunol. Ther. Exp. 2016, 64, 111. 10.1007/s00005-015-0377-3 26658771

[4] M. J. Yousefzadeh , R. R. Flores , Y. Zhu , Z. C. Schmiechen , R. W. Brooks , C. E. Trussoni , Y. Cui , L. Angelini , K.‐A. Lee , S. J. Mcgowan , A. L. Burrack , D. Wang , Q. Dong , A. Lu , T. Sano , R. D. O'Kelly , C. A. Mcguckian , J. I. Kato , M. P. Bank , E. A. Wade , S. P. S. Pillai , J. Klug , W. C. Ladiges , C. E. Burd , S. E. Lewis , N. F. Larusso , N. V. Vo , Y. Wang , E. E. Kelley , J. Huard , et al., Nature 2021, 594, 100. 33981041 10.1038/s41586-021-03547-7 PMC8684299

[5] G. L. Naert , S. Rivest , J. Mol. Cell Biol. 2013, 5, 284. 23892208 10.1093/jmcb/mjt028

[6] F. Ginhoux , S. Jung , Nat. Rev. Immunol. 2014, 14, 392. 24854589 10.1038/nri3671

[7] S. Li , E. Y. Hayden , V. J. Garcia , D.‐T. Fuchs , J. Sheyn , D. A. Daley , A. Rentsendorj , T. Torbati , K. L. Black , U. Rutishauser , D. B. Teplow , Y. Koronyo , M. Koronyo‐Hamaoui , Front. Immunol. 2020, 11, 49. 32082319 10.3389/fimmu.2020.00049 PMC7005081

[8] C. L. Hsieh , E. C. Niemi , S. H. Wang , C. C. Lee , D. Bingham , J. Zhang , M. L. Cozen , I. Charo , E. J. Huang , J. Liu , M. C. Nakamura , J. Neurotrauma 2014, 31, 1677. 24806994 10.1089/neu.2013.3252 PMC4545982

[9] J. M. Morganti , T. D. Jopson , S. Liu , L.‐K. Riparip , C. K. Guandique , N. Gupta , A. R. Ferguson , S. Rosi , J. Neurosci. 2015, 35, 748. 25589768 10.1523/JNEUROSCI.2405-14.2015 PMC4293420

[10] B. D. Semple , N. Bye , M. Rancan , J. M. Ziebell , M. C. Morganti‐Kossmann , J. Cereb. Blood Flow Metab. 2010, 30, 769. 20029451 10.1038/jcbfm.2009.262 PMC2949175

[11] P. E.­L. C. Leite , M. R. Pereira , G. Harris , D. Pamies , L. M. G. Dos Santos , J. M. Granjeiro , H. T. Hogberg , T. Hartung , L. Smirnova , Part. Fibre Toxicol. 2019, 16. 10.1186/s12989-019-0307-3 PMC6545685 31159811

[12] K. Grenier , J. Kao , P. Diamandis , Mol. Psychiatry 2020, 25, 254. 31444473 10.1038/s41380-019-0500-7

[13] D. Pamies , T. Hartung , H. T. Hogberg , Exp. Biol. Med. 2014, 239, 1096. 10.1177/1535370214537738 PMC4779552 24912505

[14] M. Jurga , A. W. Lipkowski , B. Lukomska , L. Buzanska , K. Kurzepa , T. Sobanski , A. Habich , S. Coecke , B. Gajkowska , K. Domanska‐Janik , Tissue Eng., Part C 2009, 15, 365. 10.1089/ten.tec.2008.0485 19719393

[15] H.‐Y. Tan , H. Cho , L. P. Lee , Nat. Biomed. Eng. 2021, 5, 11.33318650

[16] P. R. Ormel , R. Vieira de Sá , E. J. Van Bodegraven , H. Karst , O. Harschnitz , M. A. M. Sneeboer , L. E. Johansen , R. E. Van Dijk , N. Scheefhals , A. Berdenis Van Berlekom , E. Ribes Martã­Nez , S. Kling , H. D. Macgillavry , L. H. Van Den Berg , R. S. Kahn , E. M. Hol , L. D. De Witte , R. J. Pasterkamp , Nat. Commun. 2018, 9, 4167. 30301888 10.1038/s41467-018-06684-2 PMC6177485

[17] Z. Ao , H. Cai , Z. Wu , S. Song , H. Karahan , B. Kim , H.‐C. Lu , J. Kim , K. Mackie , F. Guo , Lab Chip 2021, 21, 2751. 34021557 10.1039/d1lc00030f PMC8493632

[18] J. Penney , W. T. Ralvenius , L.‐H. Tsai , Mol. Psychiatry 2020, 25, 148. 31391546 10.1038/s41380-019-0468-3 PMC6906186

[19] S. E. Park , A. Georgescu , D. Huh , Science 2019, 364, 960. 31171693 10.1126/science.aaw7894 PMC7764943

[20] T. Takebe , J. M. Wells , Science 2019, 364, 956. 31171692 10.1126/science.aaw7567 PMC8212787

[21] A. Sontheimer‐Phelps , B. A. Hassell , D. E. Ingber , Nat. Rev. Cancer 2019, 19, 65. 30647431 10.1038/s41568-018-0104-6

[22] F. Yu , W. Hunziker , D. Choudhury , Micromachines 2019, 10, 165. 10.3390/mi10030165 PMC6470849 30818801

[23] V. Velasco , S. A. Shariati , R. Esfandyarpour , Microsyst. Nanoeng. 2020, 6, 76. 34567686 10.1038/s41378-020-00185-3 PMC8433138

[24] Y. Shou , F. Liang , S. Xu , X. Li , Front. Cell Developmental Biol. 2020, 8, 579659. 10.3389/fcell.2020.579659 PMC7642488 33195219

[25] F. Duzagac , G. Saorin , L. Memeo , V. Canzonieri , F. Rizzolio , Cancers 2021, 13, 737. 33578886 10.3390/cancers13040737 PMC7916612

[26] I. Khan , A. Prabhakar , C. Delepine , H. Tsang , V. Pham , M. Sur , Biomicrofluidics 2021, 15, 024105. 33868534 10.1063/5.0041027 PMC8043249

[27] Y. Wang , L. Wang , Y. Zhu , J. Qin , Lab Chip 2018, 18, 851. 29437173 10.1039/c7lc01084b

[28] B. Zhang , M. Montgomery , M. Â. D. Chamberlain , S. Ogawa , A. Korolj , A. Pahnke , L. A. Wells , S. P. Massã , J. Kim , L. Reis , A. Momen , S. S. Nunes , A. R. Wheeler , K. Nanthakumar , G. Keller , M. V. Sefton , M. Radisic , Nat. Mater. 2016, 15, 669. 26950595 10.1038/nmat4570 PMC4879054

[29] H. Liu , Y. Wang , H. Wang , M. Zhao , T. Tao , X.u Zhang , J. Qin , Adv. Sci. 2020, 7, 1903739. 10.1002/advs.201903739 PMC7284190 32537414

[30] Z. Wu , Z. Gong , Z. Ao , J. Xu , H. Cai , M. Muhsen , S. Heaps , M. Bondesson , S. Guo , F. Guo , ACS Appl. Bio Mater. 2020, 3, 6273. 10.1021/acsabm.0c00768 35021758

[31] H. Wang , H. Liu , X.u Zhang , Y. Wang , M. Zhao , W. Chen , J. Qin , ACS Appl. Mater Interfaces 2021, 13, 3199. 33405509 10.1021/acsami.0c20434

[32] Z. Ao , H. Cai , Z. Wu , L. Hu , X. Li , C. Kaurich , M. Gu , L. Cheng , X. Lu , F. Guo , Theranostics 2022, 12, 3628. 35664082 10.7150/thno.71761 PMC9131272

[33] F. Guo , Y. Xie , S. Li , J. Lata , L. Ren , Z. Mao , B. Ren , M. Wu , A. Ozcelik , T. J. Huang , Lab Chip 2015, 15, 4517. 26507411 10.1039/c5lc01049g PMC4683015

[34] F. Guo , P. Li , J. B. French , Z. Mao , H. Zhao , S. Li , N. Nama , J. R. Fick , S. J. Benkovic , T. J. Huang , Proc. Natl. Acad. Sci. USA 2015, 112, 43. 25535339 10.1073/pnas.1422068112 PMC4291613

[35] F. Guo , Z. Mao , Y. Chen , Z. Xie , J. P. Lata , P. Li , L. Ren , J. Liu , J. Yang , M. Dao , S. Suresh , T. J. Huang , Proc. Natl. Acad. Sci. USA 2016, 113, 1522. 26811444 10.1073/pnas.1524813113 PMC4760790

[36] X. Ding , P. Li , S.‐C. S. Lin , Z. S. Stratton , N. Nama , F. Guo , D. Slotcavage , X. Mao , J. Shi , F. Costanzo , T. J. Huang , Lab Chip 2013, 13, 3626. 23900527 10.1039/c3lc50361e PMC3992948

[37] A. Ozcelik , J. Rufo , F. Guo , Y. Gu , P. Li , J. Lata , T. J. Huang , Nat. Methods 2018, 15, 1021. 30478321 10.1038/s41592-018-0222-9 PMC6314293

[38] Z. Ao , H. Cai , Z. Wu , J. Ott , H. Wang , K. Mackie , F. Guo , Lab Chip 2020, 21, 688. 10.1039/d0lc01141j PMC8464403 33514983

[39] H. Cai , Z. Ao , L. Hu , Y. Moon , Z. Wu , H.‐C. Lu , J. Kim , F. Guo , Analyst 2020, 145, 6243. 32840509 10.1039/d0an01373k PMC7530134

[40] H. Cai , Z. Wu , Z. Ao , A. Nunez , B. Chen , L. Jiang , M. Bondesson , F. Guo , Biofabrication 2020, 12, 035025. 32438350 10.1088/1758-5090/ab9582

[41] B. Chen , Y. Wu , Z. Ao , H. Cai , A. Nunez , Y. Liu , J. Foley , K. Nephew , X. Lu , F. Guo , Lab Chip 2019, 19, 1755. 30918934 10.1039/c9lc00135b

[42] Z. Ao , Z. Wu , H. Cai , L. Hu , X. Li , C. Kaurich , J. Chang , M. Gu , C. Liang , F. Guo , Adv. Sci. 2022, 2201478. 10.1002/advs.202201478 PMC9353481 35611994

[43] K. A. Homan , N. Gupta , K. T. Kroll , D. B. Kolesky , M. Skylar‐Scott , T. Miyoshi , D. Mau , M. T. Valerius , T. Ferrante , J. V. Bonventre , J. A. Lewis , R. Morizane , Nat. Methods 2019, 16, 255. 30742039 10.1038/s41592-019-0325-y PMC6488032

[44] M. Zhang , P. Wang , R. Luo , Y. Wang , Z. Li , Y. Guo , Y. Yao , M. Li , T. Tao , W. Chen , J. Han , H. Liu , K. Cui , X. Zhang , Y. Zheng , J. Qin , Adv. Sci. 2021, 8, 2002928. 10.1002/advs.202002928 PMC7646023 33173719

[45] M. Nikolaev , O. Mitrofanova , N. Broguiere , S. Geraldo , D. Dutta , Y. Tabata , B. Elci , N. Brandenberg , I. Kolotuev , N. Gjorevski , H. Clevers , M. P. Lutolf , Nature 2020, 585, 574. 32939089 10.1038/s41586-020-2724-8

[46] Z. Ao , H. Cai , Z. Wu , J. Krzesniak , C. Tian , Y. Y. Lai , K. Mackie , F. Guo , Anal. Chem. 2022, 94, 1365. 34928595 10.1021/acs.analchem.1c04641 PMC11483356

[47] B. Schuster , M. Junkin , S. Kashaf , I. Romero‐Calvo , K. Kirby , J. Matthews , C. Weber , A. Rzhetsk , K. White , Nat. Commun. 2020, 11, 1. 33077832 10.1038/s41467-020-19058-4 PMC7573629

[48] J. A. Brassard , M. Nikolaev , T. Hübscher , M. Hofer , M. P. Lutolf , Nat. Mater. 2021, 20, 22. 32958879 10.1038/s41563-020-00803-5

[49] N. Brandenberg , S. Hoehnel , F. Kuttler , K. Homicsko , C. Ceroni , T. Ringel , N. Gjorevski , G. Schwank , G. Coukos , G. Turcatti , M. P. Lutolf , Nat. Biomed. Eng. 2020, 4, 863. 32514094 10.1038/s41551-020-0565-2

[50] X. Qian , H. N. Nguyen , M. M. Song , C. Hadiono , S. C. Ogden , C. Hammack , B. Yao , G. R. Hamersky , F. Jacob , C. Zhong , K.‐J. Yoon , W. Jeang , L. Lin , Y. Li , J. Thakor , D. A. Berg , C. Zhang , E. Kang , M. Chickering , D. Nauen , C.‐Y. Ho , Z. Wen , K. M. Christian , P.‐Y. Shi , B. J. Maher , H. Wu , P. Jin , H. Tang , H. Song , G.‐L. Ming , Cell 2016, 165, 1238. 27118425 10.1016/j.cell.2016.04.032 PMC4900885

[51] H. Cai , Z. Ao , Z. Wu , S. Song , K. Mackie , F. Guo , Lab Chip 2021, 21, 2194. 33955446 10.1039/d1lc00145k PMC8243411

[52] S.‐J. Yoon , L. S. Elahi , A. M. Pașca , R. M. Marton , A. Gordon , O. Revah , Y. Miura , E. M. Walczak , G. M. Holdgate , H. C. Fan , J. R. Huguenard , D. H. Geschwind , S. P. Pașca , Nat. Methods 2019, 16, 75. 30573846 10.1038/s41592-018-0255-0 PMC6677388

[53] P. Arlotta , B. J. Molyneaux , J. Chen , J. Inoue , R. Kominami , J. D. Macklis , Neuron 2005, 45, 207. 15664173 10.1016/j.neuron.2004.12.036

[54] E. Gatta , A. Guidotti , V. Saudagar , D. R. Grayson , D. Aspesi , S. C. Pandey , G. Pinna , Int. J. Neuropsychopharmacol. 2021, 24, 130. 32968808 10.1093/ijnp/pyaa073 PMC7883893

[55] S. Yoo , J. Kim , P. Lyu , T. V. Hoang , A. Ma , V. Trinh , W. Dai , L. Jiang , P. Leavey , L. Duncan , J.‐K. Won , S.‐H. Park , J. Qian , S. P. Brown , S. Blackshaw , Sci. Adv. 2021, 7, 3777. 10.1126/sciadv.abg3777 PMC8163082 34049878

[56] A. Chou , K. Krukowski , J. Morganti , L.‐K. Riparip , S. Rosi , Int. J. Mol. Sci. 2018, 19, 1616. 10.3390/ijms19061616 PMC6032263 29848996

[57] J. M. Morganti , L.‐K. Riparip , A. Chou , S. Liu , N. Gupta , S. Rosi , J. Neuroinflammation 2016, 13, 80. 27090212 10.1186/s12974-016-0547-1 PMC4835854

[58] N. A. Renner , N. S. Ivey , R. K. Redmann , A. A. Lackner , A. G. Maclean , J. Neurovirol. 2011, 17, 146. 21279498 10.1007/s13365-010-0017-y PMC3086688

[59] W. L. Thompson , L. J. Van Eldik , Brain Res. 2009, 1287, 47. 19577550 10.1016/j.brainres.2009.06.081 PMC2725204

[60] C. Luo , E. Urgard , T. Vooder , A. Metspalu , Med. Hypotheses 2011, 77, 174. 21530094 10.1016/j.mehy.2011.04.002

[61] P. S. Minhas , A. Latif‐Hernandez , M. R. Mcreynolds , A. S. Durairaj , Q. Wang , A. Rubin , A. U. Joshi , J. Q. He , E. Gauba , L. Liu , C. Wang , M. Linde , Y. Sugiura , P. K. Moon , R. Majeti , M. Suematsu , D. Mochly‐Rosen , I. L. Weissman , F. M. Longo , J. D. Rabinowitz , K. I. Andreasson , Nature 2021, 590, 122. 33473210 10.1038/s41586-020-03160-0 PMC8274816

[62] E. S. Chambers , M. Vukmanovic‐Stejic , B. B. Shih , H. Trahair , P. Subramanian , O. P. Devine , J. Glanville , D. Gilroy , M. H. A. Rustin , T. C. Freeman , N. A. Mabbott , A. N. Akbar , Nat. Aging 2021, 1, 101. 10.1038/s43587-020-00010-6 37118005

[63] S. Kyrkanides , A. H. Moore , J. A. Olschowka , J. C. Daeschner , J. P. Williams , J. T. Hansen , M. Kerry Oâ Banion , Mol. Brain Res. 2002, 104, 159. 12225870 10.1016/s0169-328x(02)00353-4

[64] C. Franceschi , P. Garagnani , P. Parini , C. Giuliani , A. Santoro , Nat. Rev. Endocrinol. 2018, 14, 576. 30046148 10.1038/s41574-018-0059-4

[65] M. J. Yousefzadeh , R. R. Flores , Y. Zhu , Z. C. Schmiechen , R. W. Brooks , C. E. Trussoni , Y. Cui , L. Angelini , K.‐A. Lee , S. J. Mcgowan , A. L. Burrack , D. Wang , Q. Dong , A. Lu , T. Sano , R. D. O'Kelly , C. A. Mcguckian , J. I. Kato , M. P. Bank , E. A. Wade , S. P. S. Pillai , J. Klug , W. C. Ladiges , C. E. Burd , S. E. Lewis , N. F. Larusso , N. V. Vo , Y. Wang , E. E. Kelley , J. Huard , et al., Nature 2021, 594, 100. 33981041 10.1038/s41586-021-03547-7 PMC8684299

[66] A. D. Mooradian , Neurobiol. Aging 1988, 9, 31. 3288893 10.1016/s0197-4580(88)80013-7

[67] A. Laveskog , R. Wang , L. Bronge , L.‐O. Wahlund , C. Qiu , AJNR Am. J. Neuroradiol. 2018, 39, 70. 29170267 10.3174/ajnr.A5455 PMC7410682

[68] N. Terrando , L. I. Eriksson , J. Kyu Ryu , T. Yang , C. Monaco , M. Feldmann , M. Jonsson Fagerlund , I. F. Charo , K. Akassoglou , M. Maze , Ann. Neurol. 2011, 70, 986. 22190370 10.1002/ana.22664 PMC4556354

