# Supplementary material for: Understanding Immune‐Driven Brain Aging by Human Brain Organoid Microphysiological Analysis Platform
Source: Adv Sci (Weinh). 2022 Jul 31;9(27):2200475. doi: 10.1002/advs.202200475 (PMC9507385; doi:10.1002/advs.202200475)
Supplement: Supplementary file 1 — Supporting Information [file ADVS-9-2200475-s001.pdf]

ADVANCED SCIENCE
Open Access

Supporting Information

for *Adv. Sci.*, DOI 10.1002/advs.202200475

Understanding Immune-Driven Brain Aging by Human Brain Organoid Microphysiological Analysis Platform

Zheng Ao, Sunghwa Song, Chunhui Tian, Hongwei Cai, Xiang Li, Yifei Miao, Zhuhao Wu, Jonathan Krzesniak, Bo Ning, Mingxia Gu, Luke P. Lee and Feng Guo**

Supplementary Information

Understanding immune-driven brain aging by human brain organoid microphysiological analysis platform

Zheng Ao,[1] Sunghwa Song,[1] Chunhui Tian,[1] Hongwei Cai,[1] Xiang Li,[1] Yifei Miao,[2,3] Zhuhao Wu,[1] Jonathan Krzesniak,[1] Bo Ning,[4] Mingxia Gu,[2,3] Luke P. Lee,[5,6,7] and Feng Guo,[1]**

1. Department of Intelligent Systems Engineering, Indiana University, Bloomington, IN 47405, United States
2. Center for Stem Cell and Organoid Medicine (CuSTOM), Division of Pulmonary Biology, Division of Developmental Biology, Cincinnati Children's Hospital Medical Center, Cincinnati, OH 45229, United States
3. University of Cincinnati School of Medicine, Cincinnati, OH 45229, United States
4. Center for Cellular and Molecular Diagnostics, Department of Biochemistry and Molecular Biology, Tulane University School of Medicine, New Orleans, LA 70112, United States
5. Harvard Institute of Medicine, Harvard Medical School, Harvard University, Brigham and Women's Hospital, Boston, MA, 02115, USA
6. Department of Bioengineering, Department of Electrical Engineering and Computer Science, University of California at Berkeley, Berkeley, CA, USA.
7. Department of Biophysics, Institute of Quantum Biophysics, Sungkyunkwan University, Suwon, Korea

*Corresponding emails: lplee@bwh.harvard.edu (L.P.L.) or fengguo@iu.edu (F.G.)

Contents:

Supplementary Figure

- **Figure S1:** Device design of organoid microphysiological analysis platform.

Supplementary Discussion
- **Discussion S1:** Simulation of flow distribution on-chip.
- **Discussion S2:** Characterization of flow rate on-chip.

Supplementary Tables

- **Table S1:** Cortical organoid protocol medium compositions.
- **Table S2:** Antibody used in immunofluorescence staining.
- **Table S3:** Primer sequences for qPCR analysis.

References

Supplementary Figure

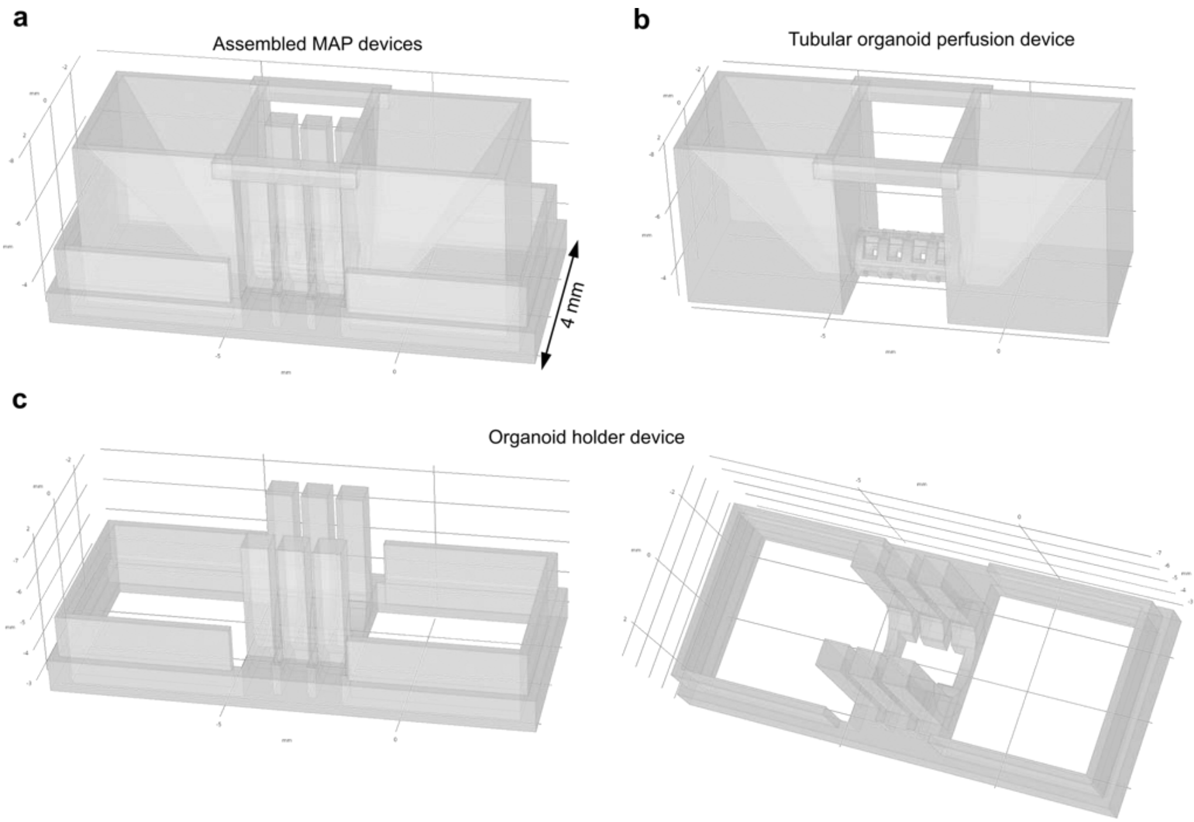

Fig.S1. Device design of organoid microphysiological analysis platform. (a) The design of assembled MAP devices with a tubular organoid perfusion device (upper) and an organoid holder device (bottom). (b) The design of tubular organoid perfusion device with a hollow and meshed tubular scaffold connected with two medium reservoirs. (c) The design of an organoid holder device on a coverslip.

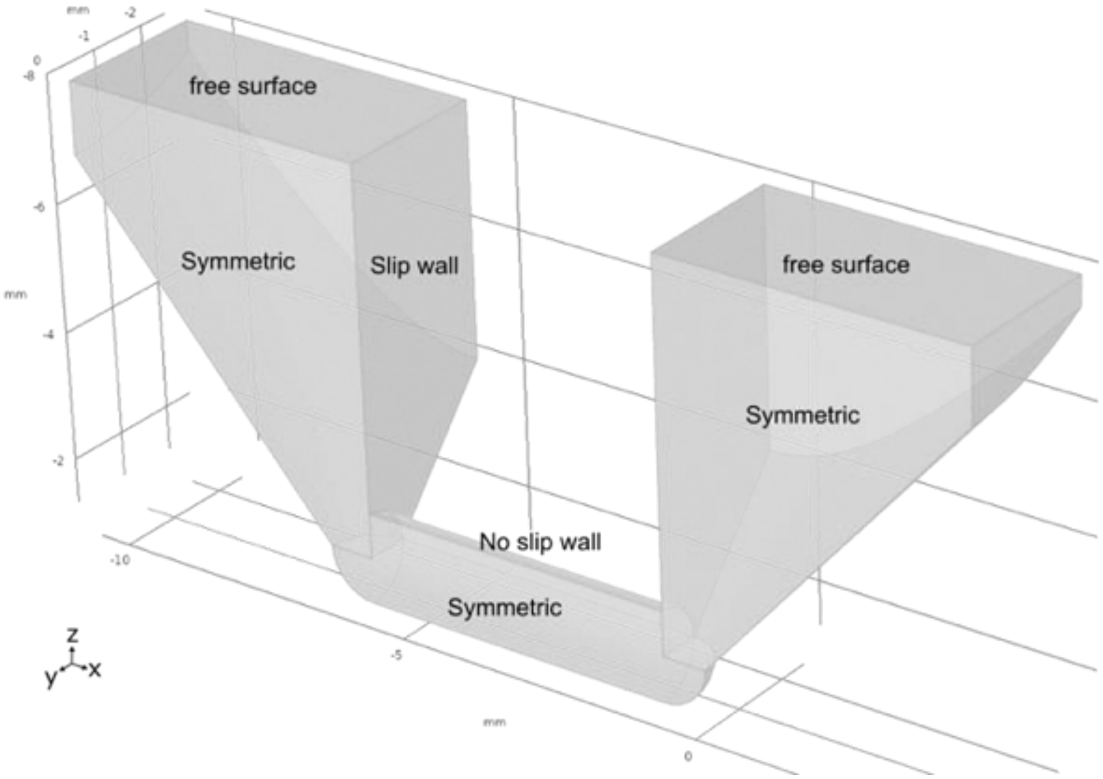

Fig.S2. Simulation domain and boundary conditions. Half liquid domain with symmetric boundary condition is simulated. The air-liquid interface is set as “free surface” condition to model the liquid movement induced by rocking. The scaffold is considered as “slip wall”, except for the tubular part is “no slip wall”.

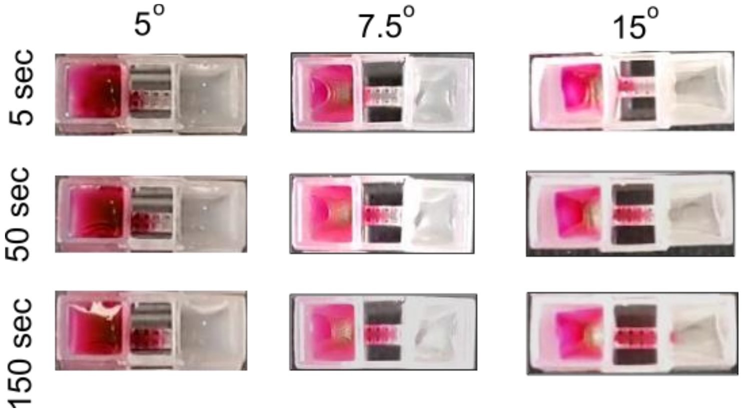

Fig. S3: **Experimental characterization of flow rate on-chip.** Representative time-lapse of one rocking cycle of the tubular device at 5º, 7.5º and 20º, respectively.

Supplementary Discussion

Discussion S1: Simulation of flow distribution on-chip. Simulation of rocking flow in the tubular device was conducted using Comsol Multiphysics (COMSOL group). To simplify the numerical simulation, only the liquid domain was considered in the simulation, while the 3D printed scaffold was consider by adding boundary condition as **Fig.S2**.

Briefly, the rocking liquid was simulated using 'laminar flow' physics module. The liquid-scaffold boundary of two side reservoir was considered as "slip wall" condition, while the tube part was considered as "no slip". To model the liquid movement, 'moving mesh' was applied to the liquid domain and the top air-liquid interface was set as 'free surface' model. To save the computational energy, half part of the model was simulated with symmetric boundary condition and quantification results were obtained using 2d model. Rocking of the fluid was simulated by periodically changed gravity. The model was solved using "Time dependent" solver.

Discussion S2. Characterization of flow rate on-chip. The average flow velocity of the device was measured by timing the passage of colored dye.[1, 2] The flow velocity was measured with five different tilting angles (5º, 7.5º, 10º, 15º and 20º) at room temperature. The rocking speed was kept as constant at 0.1 RPM. To carry out the flow visualization, the 3D printed device was placed on the rocker at a neutral position (0º tiling angle). 65 µL of culture medium was slowly added to one side of the medium reservoir using a pipette. Due to the pressure driven by the pipetting and the capillary force within the tubular channel, the medium would flow through the tubular channel and fill both medium reservoirs. 30 µL of the medium was then removed from each reservoir. Due to the surface tension, the tubular channel remained filled with medium. To minimize the disturbance of moving dye front from the pipetting, prior to the loading of colored dye, the tubular device was flash froze with liquid nitrogen for 30 sec. The flash freeze was performed by placing the device on a petri-dish, which was free-floating on liquid nitrogen within a benchtop dwar. Once the device was placed back onto the neutral-positioned rocker. 30 µL of culture medium was quickly added to both sides of the medium reservoir, with one side containing 0.83 mg/mL of Rhodamine B (ChemCruz cat# 203756). The device was then allowed to sit for 1 min at RT to let the frozen medium that was trapped within the tubular channel thaw. One rocking cycle was initiated and the corresponding flow was captured with a fixed positioned video camera on a tripod at 30 frames per second. The flow velocity within the tubular channel was quantified by timing the moving dye front passing through a predetermined distance (3mm). The video was analyzed with ImageJ and the moving dye front was determined with a fixed color intensity threshold. **Fig.S3** shows the representative time-lapse of one rocking cycle at three different tilting angles based on the video recording.

Supplementary Tables

Table S1. Medium compositions of cortical organoid protocol

Ingredients	Concentrations
**Cortical Organoid Medium I (Day 1 – Day 10)**	
DMEM/F12	1X
KOSR	15%
GlutaMax(100X)	1X
MEM-NEAA(100X)	1X
N2 supplement(100X)	1X
b-mercaptoethanol(500X)	1X
Penn/Strep(100X)	1X
SB-431542(10mM)	1µM
Dorsomorphin (10mM)	2µM
XAV939 (30mM)	2µM
**Cortical Organoid Medium II (Day 11 - Day 24)**	
DMEM/F12	0.5X
Neuralbasal	0.5X
N2 supplement(100X)	1X
B27 (wo/VA) (50X)	1X
MEM-NEAA(100X)	1X
GlutaMax(100X)	1X
Insulin (10 mg/mL)	2.5µg/mL
b-mercaptoethanol(500X)	1X
Penn/Strep(100X)	1X
EGF(500µg/mL)	
FGF-2(100µg/mL)	10ng/mL
NT3 (100µg/mL)	10ng/mL
BDNF (100µg/mL)	10ng/mL
**Cortical Organoid Medium III (Day 25+)**	
DMEM/F12	0.5X
Neuralbasal	0.5X
N2 supplement(100X)	1X
B27 (w/VA) (50X)	1X
MEM-NEAA(100X)	1X
GlutaMax(100X)	1X
Insulin (10mg/mL)	2.5µg/mL
b-mercaptoethanol(500X)	1X
Penn/Strep(100X)	1X
EGF(500µg/mL)	10ng/mL
FGF-2(100mg/mL)	10ng/mL
NT3 (100µg/mL)	10ng/mL
BDNF (100µg/mL)	10ng/mL
Ascorbic Acid (10 mg/mL)	0.2mM
cAMP (10 mM)	0.2mM
Matrigel	1%

Table S2. Antibody used in immunofluorescence staining

Antigen	Host	Vendor	Catalog#	Dilution
PAX6	Rabbit	Biolegend	901301	1:500
MAP2	Chicken	Millipore	AB5543	1:500
CDKN2A/p16INK4a	Mouse	Novus	NBP2-37736	1:300
CD11b	Rat	Thermo Fisher	14-0112-82	1:200
GFAP	Rabbit	Abcam	Ab7260	1:200

Table S3. Primer sequences for qPCR analysis

Gene	Primer sequence
hCdkn1a_Fwd	AGG TGG ACC TGG AGA CTC TCA G
hCdkn1a_Rev	TCC TCT TGG AGA AGA TCA GCC G
hCdkn2a_Fwd	CTC GTG CTG ATG CTA CTG AGG A
hCdkn2a_Rev	GGT CGG CGC AGT TGG GCT CC
hPTGES2_Fwd	CCA TCC TCT CCC TGG AAA TCT
hPTGES2_Rev	ATA TCT GCC GTC CCG AGC TT
hPTGER2_Fwd	GAC CAC CTC ATT CTC CTG GCT A
hPTGER2_Rev	AAC CTA AGA GCT TGG AGG TCC C
hPTGER4_Fwd	AGG CCA TCC GAA TTG CTT CT
hPTGER4_Rev	ACT GAG GTC TGG CAG TGA GA
hPTGS2_Fwd	CGG TGA AAC TCT GGC TAG ACA G
hPTGS2_Rev	GCA AAC CGT AGA TGC TCA GGG A
hTNFA_Fwd	CTC TTC TGC CTG CTG CAC TTT G
hTNFA_Rev	ATG GGC TAC AGG CTT GTC ACT C
hIFNG_Fwd	GAG TGT GGA GAC CAT CAA GGA AG
hIFNG_Rev	TGC TTT GCG TTG GAC ATT CAA GTC
hIL1B_Fwd	CAG AAG TAC CTG AGC TCG CC
hIL1B_Rev	AGA TTC GTA GCT GGA TGC CG
hARG1_Fwd	TCA TCT GGG TGG ATG CTC ACA C
hARG1_Rev	GAG AAT CCT GGC ACA TCG GGA A
hMCP1_Fwd	CTC TCG CCT CCA GCA TGA AA
hMCP1_Rev	GAA GAA GAG GGG GCC TTA CC
hLIF_Fwd	AGA TCA GGA GCC AAC TGG CAC A
hLIF_Rev	GCC ACA TAG CTT GTC CAG GTT G
hMCP3_Fwd	ACA GAA GGA CCA CCA GTA GCC A
hMCP3_Rev	GGT GCT TCA TAA AGT CCT GGA CC
hMCP2_Fwd	TAT CCA GAG GCT GGA GAG CTA C

hMCP2_Rev	TGG AAT CCC TGA CCC ATC TCT C
hRANTES_Fwd	TCA TTG CTA CTG CCC
hRANTES_Rev	TCG GGT GAC AAA GAC
hTOMM20_Fwd	CGA CCG CAA AAG ACG
hTOMM20_Rev	GCT TCA GCA TCT TTA
hVDAC1_Fwd	GCA AAA TCC CGA GTG
hVDAC1_Rev	TCC AGG CAA GAT TGA CAG CGG T
hSLC13a3_Fwd	CCA TTG AGG AGT GGA
hSLC13a3_Rev	GTG TTG CTC AGC CAC
hNOS2_Fwd	GCT CTA CAC CTC CAA TGT GAC C
hNOS2_Rev	CTG CCG AGA TTT GAG CCT CAT G
hGFAP_Fwd	CTG GAG AGG AAG ATT GAG TCG C
hGFAP_Rev	ACG TCA AGC TCC ACA TGG ACC T
hSERPINA3_Fwd	CCT GAA CGA CAT ACT
hSERPINA3_Rev	CAT CAA GCA CAG CCT
hVimentin_Fwd	AGG CAA AGC AGG AGT
hVimentin_Rev	ATC TGG CGT TCC AGG
hNestin_Fwd	TCA AGA TGT CCC TCA GCC TGG A
hNestin_Rev	AAG CTG AGG GAA GTC TTG GAG C
GAPDH_Fwd	GAC AGT CAG CCG CAT CTT CT
GAPDH_Rev	AAA TGA GCC CCA GCC TTC TC

References

1. Wang, Y.I. & Shuler, M.L. UniChip enables long-term recirculating unidirectional perfusion with gravity-driven flow for microphysiological systems. *Lab Chip* **18**, 2563-2574 (2018).
2. Lin, T.Y., Do, T., Kwon, P. & Lillehoj, P.B. 3D printed metal molds for hot embossing plastic microfluidic devices. *Lab Chip* **17**, 241-247 (2017).
